# Polyelectrolyte Microcapsules Enhance the Stability of β-Galactosidase Under Simulated Gastrointestinal Conditions

**DOI:** 10.3390/gels12080685

**Published:** 2026-08-03

**Authors:** Yuri S. Chebykin, Aleksandr L. Kim, Sergey A. Tikhonenko

**Affiliations:** 1 Institute of Theoretical and Experimental Biophysics Russian Academy of Science, Institutskaya St., 3, 142290 Puschino, Russia; kobepoftruth@gmail.com (Y.S.C.); tikhonenkosa@gmail.com (S.A.T.); 2 Moscow Polytechnic University (Moscow Polytech), Bolshaya Semyonovskaya Str., 38, 107023 Moscow, Russia

**Keywords:** polyelectrolyte microcapsules, β-galactosidase, lactase deficiency, enzyme stability, manganese carbonate

## Abstract

Oral enzyme replacement therapy for lactase deficiency is limited by the rapid degradation of free β-galactosidase in the gastrointestinal tract. To overcome this, β-galactosidase was encapsulated into polyelectrolyte microcapsules (PMCs) via layer-by-layer assembly using MnCO_3_ sacrificial templates, with CaCO_3_-based PMCs as a reference. MnCO_3_-PMCs achieved 99.4% encapsulation efficiency and retained 85.8% of initial activity, significantly outperforming CaCO_3_-PMCs (86.0% and 29.7%). Under simulated gastric conditions (pH 2.0, pepsin), the free enzyme and CaCO_3_-PMCs were completely inactivated, whereas MnCO3-PMCs preserved ~86% activity. In simulated intestinal fluid, MnCO_3_-PMCs exhibited a 4.3-fold activity increase within the first hour and maintained a 2.5-fold enhancement after 70 h, while the free enzyme progressively inactivated. Furthermore, MnCO_3_-PMCs demonstrated superior storage stability, retaining 64% of initial activity after 90 days at 4 °C, compared with 25% for CaCO_3_-PMCs. Although immobilization increased the Michaelis constant, the shift was smaller for MnCO_3_-PMCs (8.9-fold) than for CaCO_3_-PMCs (15.2-fold). In conclusion, MnCO_3_-templated PMCs effectively protect β-galactosidase from gastrointestinal degradation, sustain prolonged catalytic activity, and offer excellent storage stability, highlighting their strong potential for improving oral enzyme replacement therapy in lactose intolerance.

## 1. Introduction

Lactase deficiency affects up to 65–70% of the adult population worldwide, manifesting as an inability to hydrolyze lactose due to β-galactosidase deficiency [1,2]. The clinical presentation of this condition is characterized by the development of abdominal pain syndrome, flatulence, osmotic diarrhea, and nausea, resulting from the accumulation of non-hydrolyzed lactose in the intestinal lumen and its subsequent bacterial fermentation, which significantly impairs patients’ quality of life. The clinically recommended treatment strategy is oral enzyme replacement therapy, the efficacy of which has been confirmed by reduced hydrogen concentration in exhaled air and alleviation of symptoms [3,4,5]. However, the application of free enzyme forms is limited by their rapid inactivation in the acidic gastric environment (pH 1.5–3.5) under the action of pepsin, followed by proteolysis mediated by pancreatic enzymes in the intestine prior to reaching the target site of action (pH 6.0–8.0) [6,7,8].

Existing delivery systems possess significant limitations. Enteric coatings provide protection against gastric acid but fail to address the issue of rapid drug transit through the intestine, necessitating frequent administration of high doses. Chemical modification of enzymes often reduces their catalytic activity, whereas liposomal systems exhibit instability in the presence of bile salts [9,10,11]. A critical gap in current analogues is the absence of mechanisms for prolonged retention (residence) of the enzyme within the small intestinal lumen, which is essential for the effective hydrolysis of dietary lactose. Polyelectrolyte microcapsules (PMCs), fabricated via layer-by-layer assembly (LbL), represent a promising alternative owing to the possibility of creating shells with controlled permeability and the potential for functionalization to ensure mucoadhesion and extended residence time in the intestine [12,13,14,15,16].

Previously, we demonstrated the feasibility of encapsulating β-galactosidase within polyelectrolyte microcapsules (PMCs) based on calcium carbonate (CaCO_3_) [17]. However, that prior study did not include an assessment of stability in model gastrointestinal tract media containing proteases, an evaluation of long-term storage stability, or a comparison with alternative core materials. Manganese carbonate (MnCO_3_) offers several advantages over CaCO_3_: it forms a denser shell, exhibits stability in alkaline media, and better preserves particle size upon variations in ionic strength [18,19,20,21,22], which is hypothesized to enhance the preservation of the enzyme’s native conformation. The fundamental principle of this improved encapsulation method relies on the distinct physicochemical properties of the sacrificial core. Unlike highly porous CaCO_3_ spherulites, MnCO_3_ cores possess a denser, less porous structure. This minimizes the deep penetration of polyelectrolytes into the core volume during layer-by-layer assembly, thereby reducing non-specific interactions and conformational stress on the encapsulated enzyme, while its inherent buffering capacity helps maintain a favorable local microenvironment.

The aim of this study is to evaluate the functional stability of β-galactosidase encapsulated within polyelectrolyte microcapsules (PMCs) fabricated using MnCO_3_ sacrificial templates, in direct comparison with the traditional CaCO_3_-based system. Beyond the material substitution, this work addresses a critical scientific question: can a microcapsule architecture derived from a denser, less porous core provide the necessary functional stability to survive harsh, physiologically relevant gastrointestinal conditions (specifically pH 2.0 with pepsin, and prolonged exposure to simulated intestinal fluid with proteases) and long-term storage, where the CaCO_3_-based system fails? The novelty of this work lies in the first comprehensive empirical validation of this delivery system under these specific physiological stressors, establishing the MnCO_3_-based PMCs as a functionally viable platform for oral enzyme replacement therapy.

## 2. Results and Discussion

Building upon our previous demonstration of β-galactosidase encapsulation on CaCO_3_ cores [17], several critical challenges must be addressed for practical therapeutic application. Specifically, prior work did not account for the presence of proteolytic enzymes or physiologically relevant salt concentrations, which are essential for preserving enzymatic activity in the acidic gastric (pH 1.5–3.5) and neutral-to-alkaline intestinal (pH 6.0–8.0) environments [23,24]. Furthermore, long-term storage stability is a prerequisite for commercialization [23,25]. To address these gaps, we evaluated an alternative core material, MnCO_3_, which offers distinct advantages: formation of a denser shell [26], stability in alkaline media (up to pH 12.5), distinct buffering capacity [18], and better preservation of particle size upon variations in ionic strength [26].

In this work, β-galactosidase was encapsulated within MnCO_3_-based PMCs for the first time, and a direct comparison with CaCO_3_-based PMCs was performed with respect to key parameters: encapsulation efficiency, enzymatic activity, Michaelis–Menten kinetic parameters, stability in model gastric and intestinal solutions (under static and dynamic conditions), and long-term storage stability.

**Figure 1:**
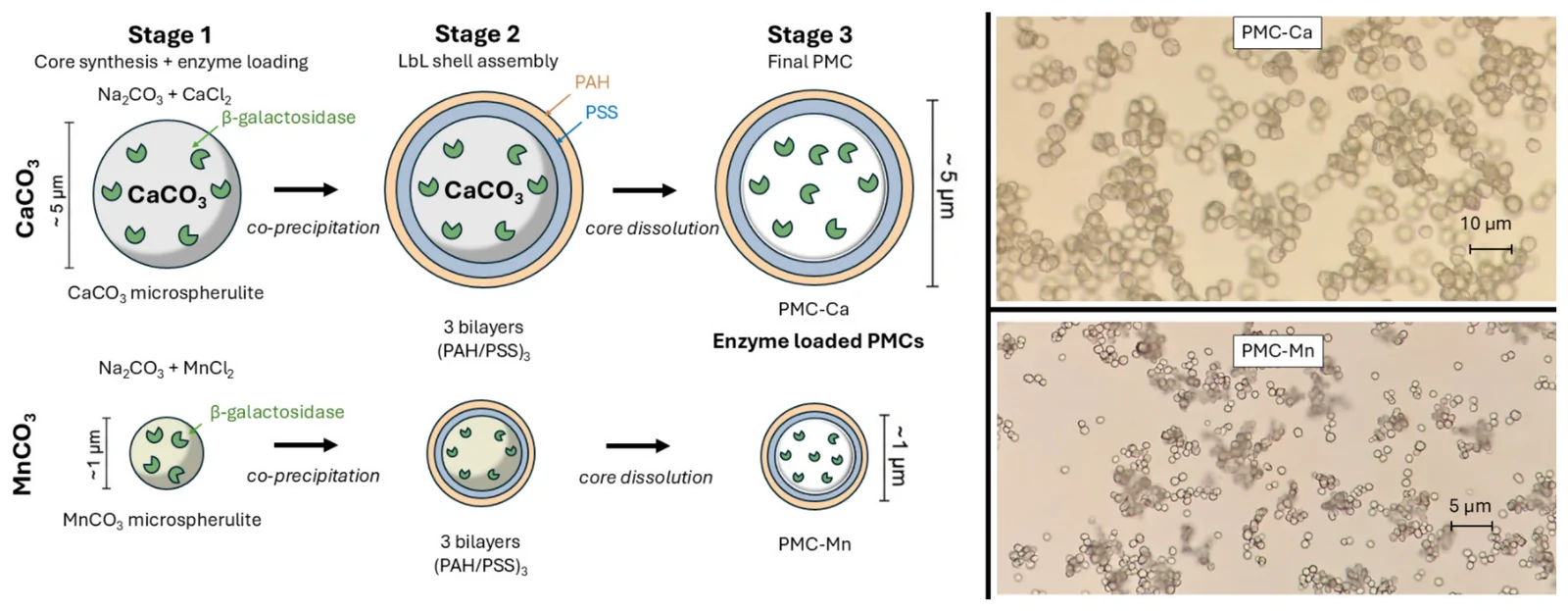
Schematic illustration of PMC fabrication. Abbreviations: LbL, layer-by-layer; PMC, polyelectrolyte microcapsule; PAH, poly(allylamine hydrochloride); PSS, sodium poly(styrenesulfonate).

In the first stage of the study, β-galactosidase was encapsulated within polyelectrolyte microcapsules (PMCs) fabricated using two distinct types of cores: calcium carbonate (CaCO_3_) and manganese carbonate (MnCO_3_). Polyelectrolyte shells were formed around the inorganic cores via layer-by-layer assembly (LbL), followed by core dissolution to yield microcapsules containing the enzyme. The PMC fabrication protocol is schematically illustrated below (Figure 1).

PMCs based on MnCO_3_ (PMCs-Mn) exhibited a size of 1 µm, whereas PMCs based on CaCO_3_ (PMCs-Ca) measured 5 µm; both populations demonstrated a normal size distribution without aggregation.

**Figure 2:**
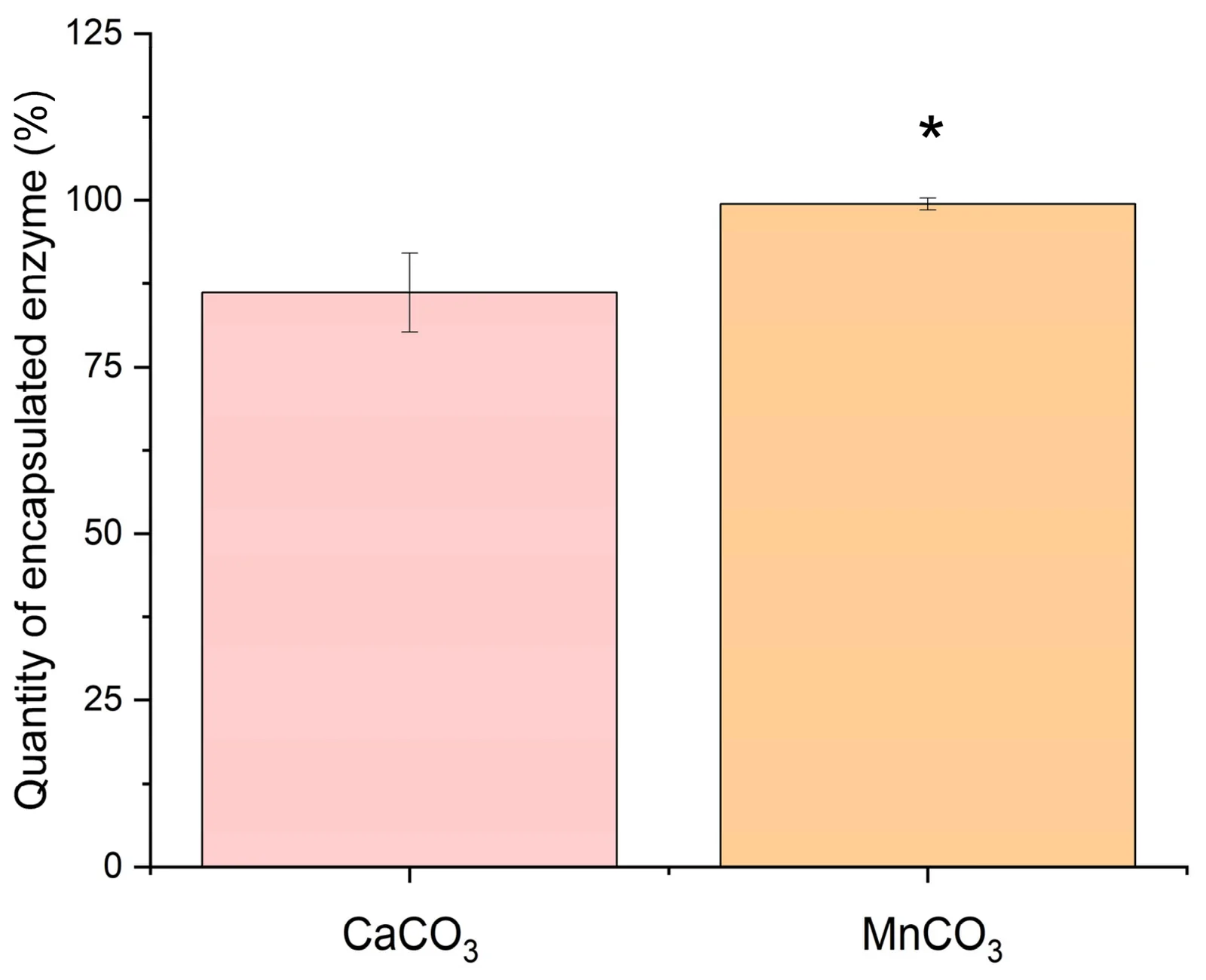
Encapsulation efficiency of β-galactosidase in polyelectrolyte microcapsules. Comparison of two types of microcapsules formed on CaCO_3_ and MnCO_3_ cores. Data are presented as mean ± SD (n = 17). The asterisk (*) indicates a statistically significant difference.

The encapsulation efficiency of β-galactosidase was evaluated for both types of microcapsules to determine the optimal core material for enzyme loading. The results are presented in Figure 2.

As shown in Figure 2, the encapsulation efficiency of β-galactosidase within MnCO_3_-based PMCs (PMCs-Mn) (99.4 ± 0.88%) was significantly higher than that within CaCO_3_-based PMCs (PMCs-Ca) (86.0 ± 5.9%) (Tukey’s post-hoc test, *p* < 0.05). The increased encapsulation percentage enables higher specific enzymatic activity per unit mass of the carrier, which directly influences the therapeutic efficacy of the system. This superior encapsulation efficiency is attributed to the fundamental differences in core formation. During co-precipitation, CaCO_3_ rapidly forms a highly porous, spongy spherulite structure, which inherently leads to partial enzyme loss or poor entrapment from the very beginning [27]. In contrast, MnCO_3_ co-precipitation yields a significantly denser, less porous core. This dense architecture physically traps the enzyme more effectively during initial particle formation, preventing early leakage and ensuring near-quantitative loading.

**Figure 3:**
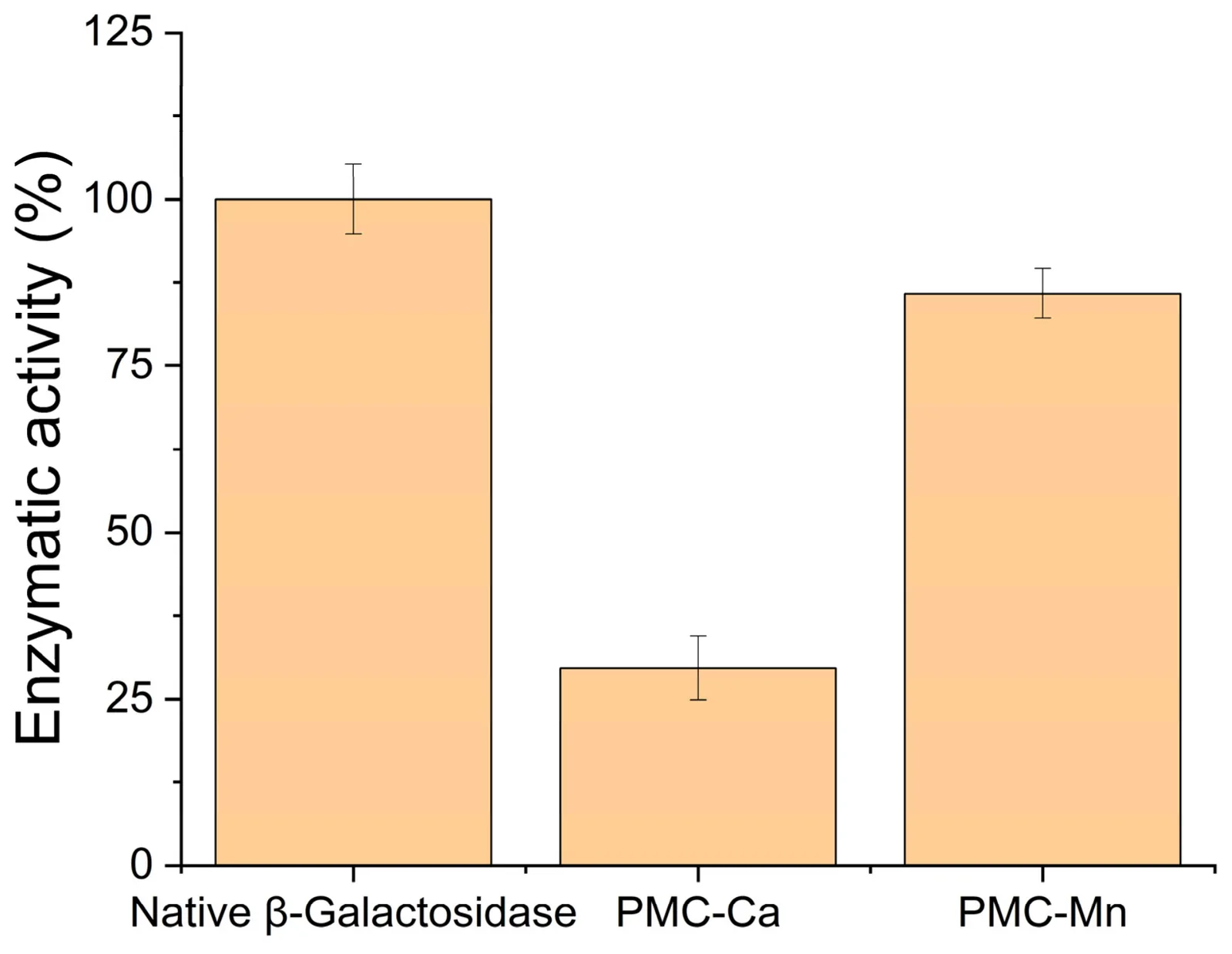
Enzymatic activity of β-galactosidase in free form and after encapsulation within CaCO_3_-based and MnCO_3_-based PMCs. Activity was normalized to an equivalent amount of enzyme (U). Reaction conditions: lactose substrate 146 mM, pH 6.0, temperature 24 °C. Glucose detection was performed using an enzymatic assay: glucose oxidase 6 mg/mL (180 U/mg), oximetric detector, potential +100 mV. The activity of the free enzyme was taken as 100%.

To assess the functional integrity of the enzyme following encapsulation, the specific activity of β-galactosidase within PMCs was measured relative to the free enzyme at equivalent protein quantities. The results are presented in Figure 3.

As shown in Figure 3, the enzymatic activity of β-galactosidase encapsulated within PMCs-Ca amounted to 29.7 ± 4.8% of the activity of the free enzyme, whereas for PMCs-Mn, this parameter reached 85.8 ± 3.7%. The reduced activity observed in PMCs-Ca is likely attributable to conformational changes in β-galactosidase during polyelectrolyte shell formation on the mesoporous CaCO_3_ spherulites. Specifically, the high specific surface area and developed porosity of this template facilitate polyelectrolyte penetration into the particle volume. This enhances non-specific interactions with the enzyme, increasing the risk of partial denaturation or steric shielding of active sites [28]. In the case of PMCs-Mn, the denser packing of polyelectrolyte layers may limit substrate access to active sites; however, it simultaneously ensures better preservation of the enzyme’s native conformation. The high retention of activity in PMCs-Mn represents a critical parameter for therapeutic enzyme delivery systems, as it determines the effectiveness of lactose hydrolysis under physiological conditions.

**Figure 4:**
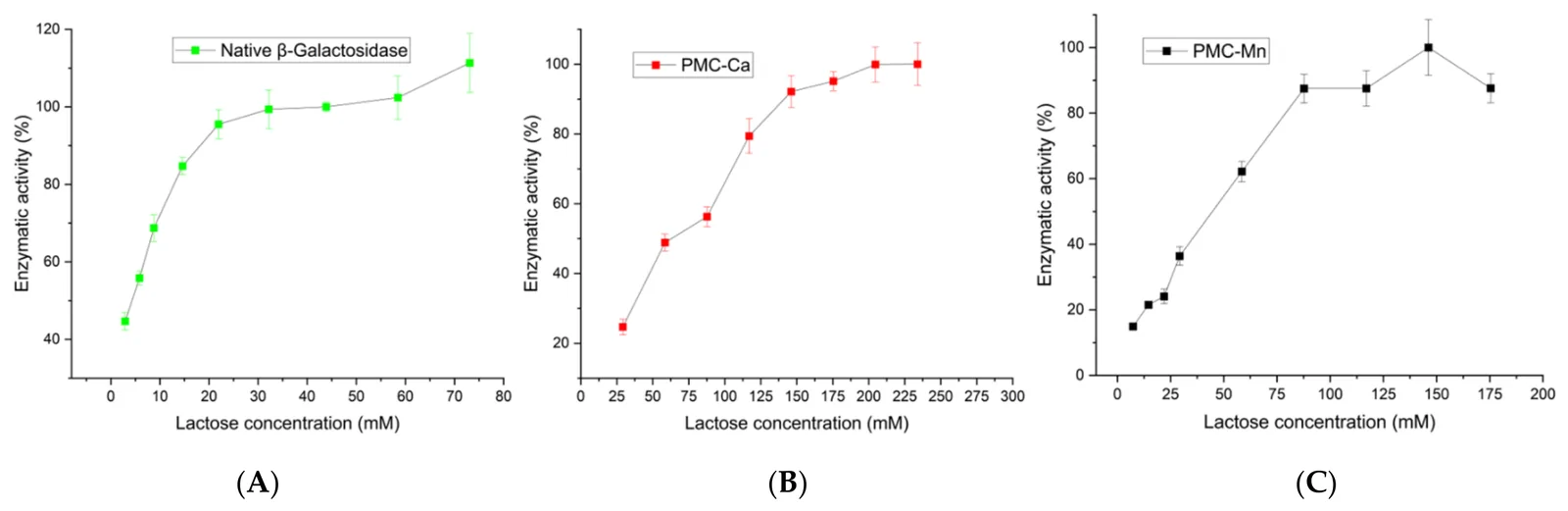
Dependence of enzymatic activity on lactose concentration. (**A**)—native β-galactosidase, (**B**)—PMCs-Ca, (**C**)—PMCs-Mn. Reaction conditions: pH 6.0, temperature 24 °C. Glucose detection: glucose oxidase 5 mg/mL (180 U/mg), oximetric detector, potential +100 mV.

To evaluate the effect of encapsulation on the kinetic properties of the enzyme, the Michaelis constants (Km) of β-galactosidase within CaCO_3_-based and MnCO_3_-based PMCs were determined in comparison with the free enzyme. Determination of Km allows assessment of enzyme-substrate affinity and identification of potential alterations in catalytic properties resulting from immobilization within the polyelectrolyte shell, which is critically important for predicting the efficiency of lactose hydrolysis under physiological conditions. The results of the kinetic analysis are presented in Figure 4.

**Figure 5:**
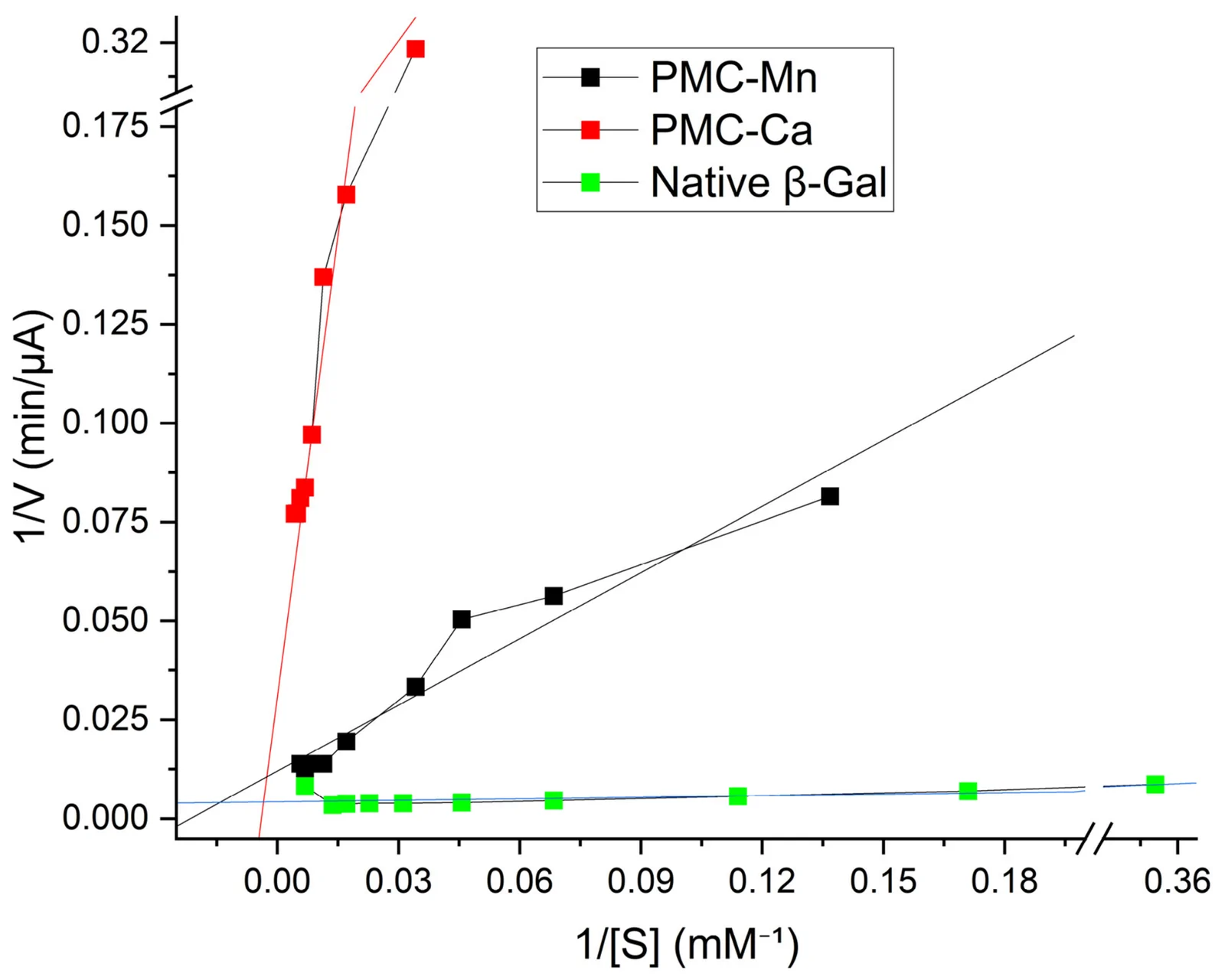
Kinetic analysis of β-galactosidase in Lineweaver–Burk coordinates. Reaction conditions: pH 6.0, temperature 24 °C. Glucose detection: glucose oxidase 5 mg/mL (180 U/mg), oximetric detector, potential +100 mV. The lines represent the linear fit for each data group.

As shown in Figure 4, encapsulation of β-galactosidase resulted in alterations to the kinetic characteristics of the enzyme, with the extent of these changes being substantially dependent on the type of microcapsules. The Michaelis constant (Km) for the native enzyme was 4.2 mM, whereas for the encapsulated forms it increased to 37.3 mM for PMCs-Mn (an 8.9-fold increase) and to 63.9 mM for PMCs-Ca (a 15.2-fold increase) (calculated using standard graphical and Lineweaver–Burk methods, as detailed in Section 4.4). The elevation in Km indicates a reduced affinity of the enzyme for its substrate, which is characteristic of the emergence of diffusion limitations upon encapsulation—the substrate must overcome the barrier presented by the polyelectrolyte shell to access the enzyme’s active sites [13]. To test this hypothesis, kinetic data were analyzed using Lineweaver–Burk coordinates (Figure 5).

Analysis of the Lineweaver–Burk plots (Figure 5) revealed that the regression lines for the free and encapsulated enzyme did not intersect at a single point, indicating distinct mechanisms of limitation for PMCs-Mn and PMCs-Ca. The line corresponding to PMCs-Mn exhibited a moderately increased slope with a minimal shift in the y-intercept, which is characteristic of predominantly diffusion-limited kinetics. In contrast, the line for PMCs-Ca demonstrated a substantially increased slope and a pronounced shift in the y-intercept, suggesting a combination of diffusion and conformational limitations. Presumably, the more developed porosity of CaCO_3_ spherulites facilitates the penetration of polyelectrolytes into the particle volume, enhancing their interactions with the enzyme and contributing to partial restriction of β-galactosidase conformational mobility. This, together with alterations in the local microenvironment of active sites, accounts for the lower residual activity observed in PMCs-Ca despite comparable diffusion barriers.

Thus, the architecture of the carrier represents a critical factor determining the kinetic characteristics of the encapsulated enzyme: capsules PMCs-Mn preserve up to 86% of the native enzyme activity, whereas PMCs-Ca lead to significant activity loss due to conformational constraints.

**Figure 6:**
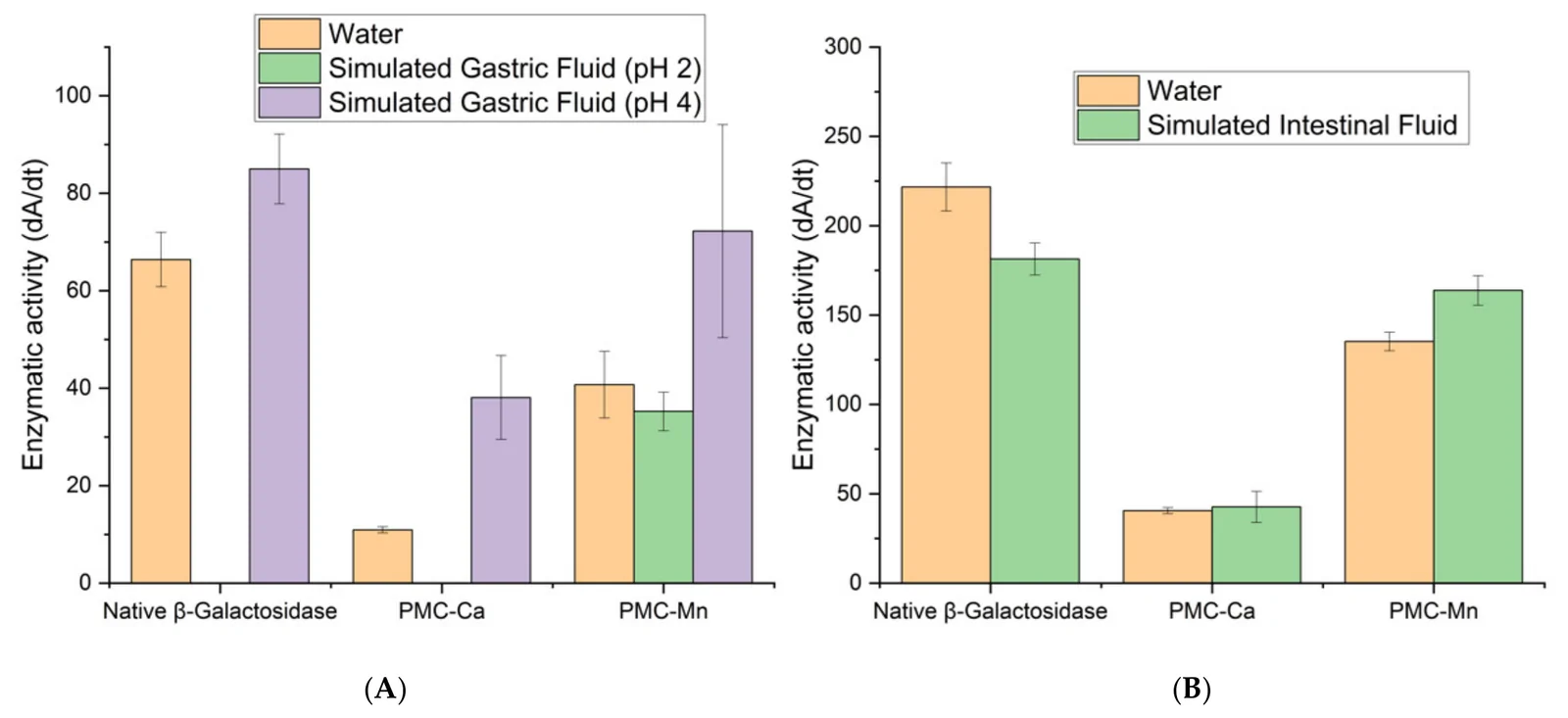
Stability of β-galactosidase in free form and after encapsulation within PMCs-Ca and PMCs-Mn under model gastrointestinal tract conditions. (**A**)—simulated gastric fluid (SGF); (**B**)—simulated intestinal fluid (SIF). Enzyme activity is expressed as the rate of current change (dA/dt, µA/min), directly proportional to oxygen consumption.

In the next stage of the study, the stability and functional activity of PMCs-Ca and PMCs-Mn were evaluated under model gastrointestinal tract conditions. For oral enzyme formulations, preservation of activity in the aggressive gastric environment (pH 2 and 4, pepsin) and subsequent functionality in the intestinal environment (pH 6.0, pancreatic enzymes, bile salts) are critically important. Experiments involving incubation of microcapsules in model gastric and intestinal solutions were performed, followed by measurement of residual enzymatic activity. The results are presented in Figure 6.

As shown in Figure 6, incubation under model gastrointestinal tract conditions exerted differential effects on enzymatic activity depending on the type of microcapsules. The activity of β-galactosidase, both free and encapsulated within PMCs-Ca and PMCs-Mn, was measured in simulated gastric fluid (SGF) at pH 2 and pH 4, as this acidity range corresponds to physiological norms. The activity of the free enzyme in SGF at pH 2 decreased to zero, consistent with results from previous experiments [17], whereas activity at pH 4 increased by 27%. In the case of PMCs-Ca, zero activity was also observed in SGF at pH 2; however, at pH 4, a 3.8-fold increase in activity was detected. PMCs-Mn exhibited a distinct pattern: in SGF at pH 2, activity decreased only slightly (by 14%) relative to the control in water at pH 6, whereas at pH 4, a substantial 75% increase in activity was observed. This stark contrast with the complete inactivation of PMCs-Ca highlights the mechanistic significance of the core architecture: the dense MnCO_3_-derived shell effectively shields the enzyme from pepsin and acidic denaturation, whereas the porous CaCO_3_-derived structure fails to provide this critical protection.

Following incubation in simulated intestinal fluid (SIF), the activity of the free enzyme decreased by 19 ± 3% compared with the initial value, indicating a detrimental effect of intestinal medium components on β-galactosidase stability. For PMCs-Ca, no significant changes in activity were detected (difference within 2 ± 1%, *p* > 0.05), suggesting a protective effect of the polyelectrolyte shell. For PMCs-Mn, an 18 ± 4% increase in activity relative to the initial level was observed. The obtained data indicate that PMCs-Mn containing encapsulated β-galactosidase maintains stability and enzymatic activity under conditions simulating both gastric (SGF) and intestinal (SIF) environments, underscoring their potential for application in enzyme replacement therapy. Regarding PMCs-Ca, it was established that the encapsulated enzyme loses activity at pH 2.0 but retains it at pH 4.0 and in SIF; this permits their use, albeit under more restricted conditions and with reduced catalytic efficiency.

One of the advantages of polyelectrolyte microcapsules for enzyme replacement therapy is their presumed capacity for intestinal retention and long-term preservation of functional activity. However, the realization of this advantage requires ensuring the stability and catalytic activity of β-galactosidase within PMCs under conditions simulating the intestinal environment over an extended period.

**Figure 7:**
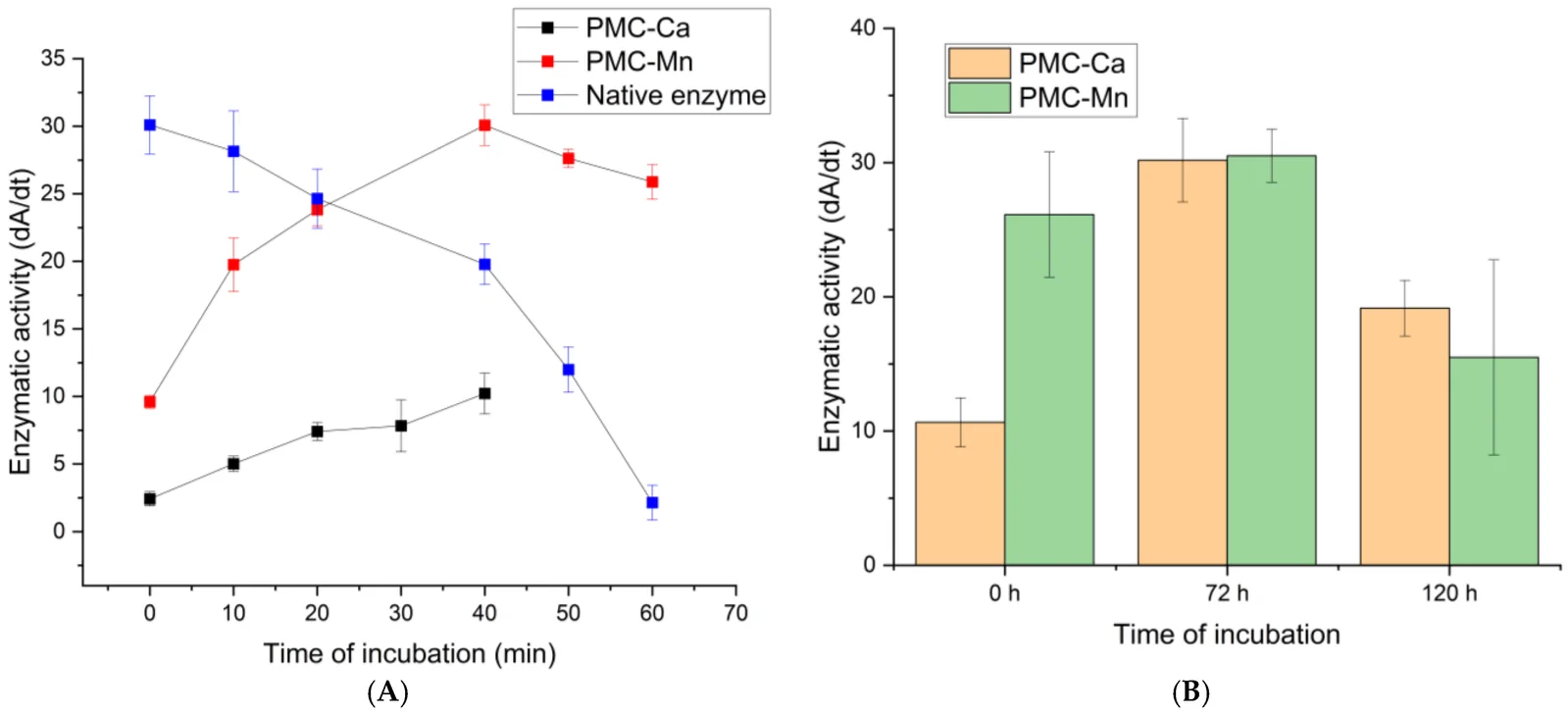
Long-term stability of β-galactosidase after encapsulation within PMCs-Ca and PMCs-Mn in simulated intestinal fluid. Incubation at pH 6.0, 37 °C. (**A**) Enzymatic activity of free and encapsulated β-galactosidase during the first 60 min of incubation in SIF; (**B**) Long-term enzymatic activity in SIF.

The intestinal environment poses a significant challenge to protein-based therapeutics due to the continuous presence of pancreatic proteases (trypsin, chymotrypsin, and elastase within pancreatin) and bile salts, which can progressively degrade free β-galactosidase and impair its catalytic function over time. Furthermore, since polyelectrolyte microcapsules are designed to prolong intestinal residence, the encapsulated enzyme must maintain structural integrity and activity despite prolonged exposure to these aggressive components. To evaluate the long-term functionality of encapsulated β-galactosidase under physiologically relevant conditions, the kinetics of enzymatic activity of PMCs-Ca and PMCs-Mn were studied in simulated intestinal fluid during prolonged incubation. This investigation allows assessment of the protective capacity of the polyelectrolyte shell against proteolytic degradation and ionic/biliary stress. The results are presented in Figure 7.

As shown in Figure 7A, during the first hour of incubation in simulated intestinal fluid, a gradual increase in enzymatic activity was observed: for PMCs-Ca, activity increased 6.6-fold relative to the initial value, whereas for PMCs-Mn, a 4.3-fold increase was detected. This gradual increase in activity is attributed to the favorable local buffering capacity of the MnCO_3_-derived microenvironment [18], combined with the time-dependent equilibration of substrate diffusion through the polyelectrolyte shell at the specific pH and ionic strength of the SIF. In sharp contrast, free β-galactosidase lost ~93% of its initial activity within the same 60-min period, retaining only ~7% of activity, which underscores the protective effect of the polyelectrolyte shell against proteolytic degradation in the intestinal milieu. The high retention of the enzyme within the capsules is further supported by our previous study [17], which demonstrated that β-galactosidase release from co-precipitation-based PMCs does not exceed 6% at pH 6.0–8.0 over 120 h. This minimal leakage, combined with the sustained activity in the presence of proteases observed here (Figure 7), confirms that the enzyme remains securely encapsulated and protected.

Given that the ultimate design goal for such microcapsules is prolonged intestinal residence (which requires future in vivo validation), it is a necessary prerequisite to first demonstrate their long-term stability under simulated conditions. Therefore, an extended stability study was conducted to evaluate this potential (Figure 7B). The results demonstrated that for PMCs-Ca, a decline in activity was observed after 70 h of incubation, whereas for PMCs-Mn, activity further increased 2.5-fold over the same period. Upon further prolongation of incubation time, activity decreased twofold in both cases, which may indicate gradual enzyme degradation.

**Figure 8:**
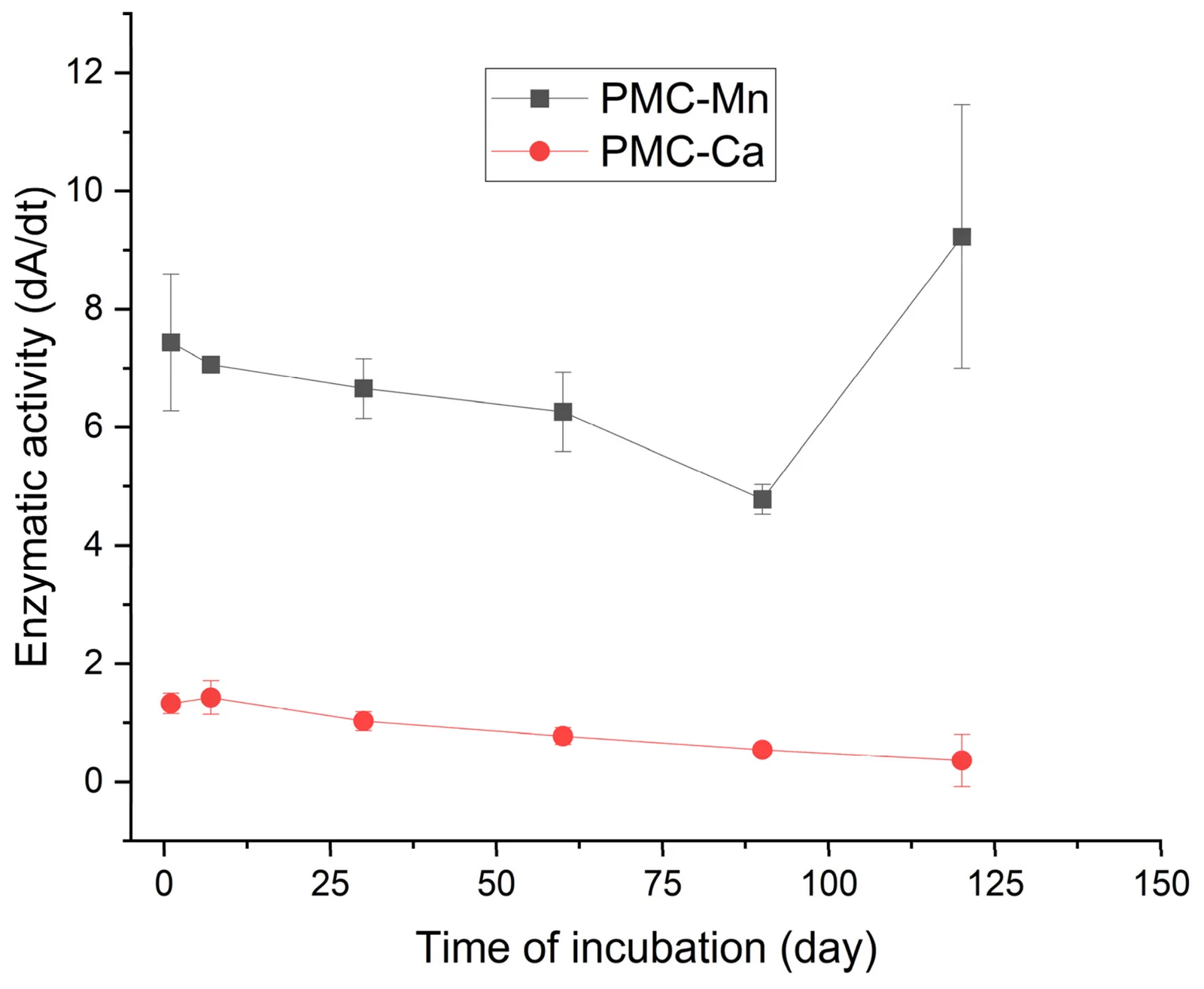
Long-term stability of β-galactosidase in free form and after encapsulation within PMCs-Ca and PMCs-Mn during storage in water. Storage conditions: 4 °C, 90 days.

To evaluate the potential for long-term storage of encapsulated β-galactosidase, the stability of PMCs-Ca and PMCs-Mn in water at 4 °C was investigated over a period of 90 days. This study allows exclusion of gradual enzyme degradation as the cause of reduced activity during prolonged incubation in model gastrointestinal solutions and determines the suitability of microcapsules for storage as an aqueous suspension. The results are presented in Figure 8.

As shown in Figure 8, storage in water at 4 °C over a period of 90 days resulted in a gradual decline in the enzymatic activity of encapsulated β-galactosidase. For PMCs-Mn, activity decreased from 7.4 ± 1.2 to 4.7 ± 0.3 µM/min, whereas for PMCs-Ca, it decreased from 1.34 ± 0.17 to 0.34 ± 0.44 µM/min. The superior retention of activity observed for PMCs-Mn indicates enhanced enzyme stability within these microcapsules during prolonged storage, confirming that significant enzyme degradation is not the primary cause of reduced activity during extended incubation in model gastrointestinal solutions.

Comparative analysis of the characteristics of polyelectrolyte microcapsules based on manganese carbonate (PMCs-Mn) and calcium carbonate (PMCs-Ca) demonstrated that PMCs-Mn provides superior performance across key parameters of β-galactosidase encapsulation. Encapsulation efficiency for PMCs-Mn reached 99.4 ± 0.88% compared with 86.0 ± 5.9% for PMCs-Ca; retention of enzymatic activity was 85.8 ± 3.7% versus 29.7 ± 4.8%, respectively; and the increase in the Michaelis constant (Km) relative to the free enzyme was less pronounced for PMCs-Mn (an 8.9-fold increase versus a 15.2-fold increase). In simulated intestinal fluid, the activity of β-galactosidase within PMCs-Mn increased by 18 ± 4%, whereas a 19 ± 3% decrease was observed for the free enzyme. Following storage of suspensions at 4 °C for 90 days, residual activity in PMCs-Mn was maintained at 64%, compared with 25% for PMCs-Ca. Based on the obtained data, PMCs-Mn can be recommended as a more promising carrier for the development of oral enzyme formulations, particularly for the delivery of β-galactosidase aimed at the correction of lactase deficiency.

## 3. Conclusions

The present study demonstrates that polyelectrolyte microcapsules fabricated on MnCO_3_ cores via layer-by-layer assembly possess substantial advantages over CaCO_3_-based analogues for the oral delivery of β-galactosidase. MnCO_3_-based microcapsules provided an encapsulation efficiency of 99.4% compared with 86.0% for CaCO_3_-based systems, and preserved enzymatic activity at 85.8% versus 29.7%, respectively. Kinetic analysis revealed a less pronounced increase in the Michaelis constant (Km) for the MnCO_3_-based system (an 8.9-fold increase versus a 15.2-fold increase relative to the native enzyme). Prolonged incubation (70 h) in simulated intestinal fluid demonstrated a 2.5-fold increase in activity for MnCO_3_-based microcapsules, whereas CaCO_3_-based capsules exhibited a decline in activity, and the free enzyme underwent progressive inactivation. Storage stability at 4 °C over 90 days was also superior for PMCs-Mn (64% residual activity versus 25% for PMCs-Ca).

The aforementioned characteristics—high loading capacity, preservation of kinetic properties, resistance to proteolysis, and storage stability—address the key limitations of current strategies for enzyme replacement therapy in lactase deficiency. The collective data indicate the promise of manganese carbonate as a core material for the development of oral enzyme formulations based on β-galactosidase encapsulated within PMCs, aimed at the correction of lactase deficiency. While the current in vitro data demonstrate potential, future research will focus on in vivo validation and optimization of mucoadhesive functionalization to prolong intestinal residence time and maximize therapeutic efficacy.

## 4. Materials and Methods

### 4.1. Materials and Reagents

Polyelectrolytes: sodium poly(styrenesulfonate) (PSS) and poly(allylamine hydrochloride) (PAH) with a molecular weight of 70 kDa were purchased from Merck (St. Louis, MO, USA). The residual monomer content was less than 10%. β-Galactosidase was obtained from Huanwei Biotech (Shijiazhuang, China). Glucose oxidase (150 U/mg), bile extract porcine, pancreatin and pepsin were purchased from Merck (St. Louis, USA). Lactose, sodium chloride, sodium hydroxide, and hydrochloric acid were supplied by Reakhim (Moscow, Russia). The purity of each chemical compound was at least 99.9%. Ethylenediaminetetraacetic acid (EDTA) was used for the dissolution of inorganic cores.

### 4.2. Synthesis of Inorganic Cores and Enzyme Encapsulation

Polyelectrolyte microcapsules (PMCs) were fabricated via layer-by-layer assembly (LbL) on sacrificial templates composed of calcium carbonate (CaCO_3_) [28] and manganese carbonate (MnCO_3_).

Synthesis of CaCO_3_ cores: CaCO_3_ microspherulites (average diameter 5.0 ± 0.5 µm) were obtained via a co-precipitation technique, following the protocol detailed in our previous studies [17,28]. Briefly, 1.5 mL of 0.33 M Na_2_CO_3_ was rapidly introduced into 1.5 mL of 0.33 M CaCl_2_ supplemented with β-galactosidase (6 mg/mL) under continuous vigorous agitation. The formed precipitate was isolated by centrifugation (500× *g*), the liquid phase was discarded, and the collected cores were immediately utilized for shell assembly.

Synthesis of MnCO_3_ cores: Manganese carbonate microparticles (mean size 1.0 ± 0.3 µm) were synthesized using an analogous co-precipitation method, with calcium chloride replaced by manganese chloride (MnCl_2_) of the same molarity.

Shell formation: The multilayer shell was constructed via the layer-by-layer (LbL) deposition of PSS (polyanion) and PAH (polycation). Core-enzyme complexes were sequentially immersed in 2 mg/mL polymer solutions (prepared in 0.5 M NaCl). Following each adsorption step, the particles were triple-washed with 2 mL of 0.5 M NaCl to eliminate unbound polymers. The final shell architecture consisted of three bilayers, terminating with a PSS outer layer, i.e., (PAH/PSS)_3_.

Core removal:

The inorganic cores were degraded by treating the particles with 20 mL of 0.2 M EDTA (pH 7.0) for 2 h. The resulting PMCs were then thoroughly rinsed three times with bidistilled water (2 mL). To evaluate the hydrodynamic diameter and particle concentration of the microcapsules, dynamic light scattering analysis was conducted using a Zetasizer Nano ZS system (Malvern Instruments, Malvern, UK)

Polyelectrolyte microcapsules formed on calcium carbonate are hereinafter referred to as PMCs-Ca, whereas those formed on manganese carbonate are designated as PMCs-Mn. In both cases, the inorganic core was removed using EDTA.

### 4.3. Measurement of β-Galactosidase Activity

Enzyme activity was assessed based on the quantity of substrate molecules converted into reaction products per unit time. β-Galactosidase activity was measured using a sequential enzymatic reaction with glucose oxidase. Glucose oxidase was added in excess (6 mg/mL, 180 U/mg) to ensure that glucose oxidation did not limit the reaction rate.

Measurements were performed by monitoring changes in dissolved oxygen concentration associated with the activity of β-galactosidase added to the reaction cell. The rate of the enzymatic reaction was monitored using a Clark-type oxygen electrode (model “Expert DK-1”, Econics Expert, Moscow, Russia) in a 1 mL cuvette. The composition of the reaction mixture for the sequential enzymatic assay was as follows: glucose oxidase 5 mg/mL, lactose 146 mM, and β-galactosidase (either free or incorporated into PMCs at a quantity of 2.5 × 10^6^ microcapsules). Standard reaction conditions: pH 6.0, temperature 24 °C. Glucose detection was performed using an oximetric method at a potential of +100 mV. The specificity of the assay was confirmed during method validation: control measurements performed without the addition of lactose yielded zero background signal, ruling out any interference from capsule components or residual ions.

In this setup, enzyme activity was recorded as the rate of change in current (dA/dt) of the oximetric detector, expressed in microamperes per minute (µA/min). This parameter is directly proportional to the rate of dissolved oxygen consumption, which in turn directly reflects the catalytic activity of β-galactosidase in the coupled enzymatic reaction.

### 4.4. Determination of Kinetic Parameters (K_m_)

To assess how encapsulation alters the kinetic behavior of the enzyme, the Michaelis constant (Km) was evaluated for both free and encapsulated β-galactosidase across a gradient of lactose concentrations. To ensure robust kinetic profiling, the parameters were derived using two complementary analytical approaches. First, a direct graphical analysis was employed to identify the precise substrate concentration corresponding to half of the maximal reaction velocity (Vmax), adhering to the standard protocols of the International Union of Biochemistry and Molecular Biology (IUBMB). Second, the experimental data were linearized using Lineweaver–Burk double-reciprocal coordinates (plotting 1/V against 1/[S]), allowing the Km value to be independently calculated from the *x*-axis intercept (−1/Km).

### 4.5. Simulation of Gastrointestinal Tract Conditions

To evaluate the stability of PMCs, model gastric and intestinal media were prepared according to the recommendations of the United States Pharmacopeia (USP):

Simulated gastric fluid (SGF): 2 g NaCl + 7 mL of 6 M HCl per 1 L of solution (pH 2 and 4). To this solution, 20 g of pepsin (2 MU) was added.

Simulated intestinal fluid (SIF): 8.6 g KH_2_PO_4_ + 77 mL of 0.2 M NaOH per 1 L of solution (pH 6.0–8.0). Bile extract porcine and pancreatin were also added to this solution.

Microcapsules were incubated in the model solutions at 37 °C. Residual enzymatic activity was measured following incubation in water. To assess long-term functionality, extended incubation (up to 70 h) was performed in the intestinal model solution (pH 6.8, 37 °C).

### 4.6. Storage Stability Studies

To evaluate the potential for long-term storage of encapsulated β-galactosidase, the stability of PMCs-Ca and PMCs-Mn in water at 4 °C was investigated over a period of 90 days. Enzyme activity was measured periodically throughout the storage period.

### 4.7. Statistical Analysis

All experimental assays were performed with a minimum of five independent replicates, except for kinetic analyses which included 17 replicates. Data are expressed as mean ± standard deviation. Statistical comparisons were executed using one-way analysis of variance (ANOVA) coupled with Tukey’s post-hoc test for multiple comparisons. A *p*-value of less than 0.05 was set as the threshold for statistical significance. All computational and graphical analyses were carried out utilizing OriginPro 2024 software.

## Data Availability

Data is contained within the article.
